# Commentary on PURPOSE-1 and PURPOSE-2 trials of lenacapavir: a breakthrough in HIV prevention

**DOI:** 10.1097/MS9.0000000000005097

**Published:** 2026-05-07

**Authors:** Mustabshira Idrees, Hafsa Iftikhar, Fatima Sohail, Mustabshirah Jadoon, Muhammad Hanif Khan

**Affiliations:** a KMU IMS Kohat, Kohat, Pakistan; b Jinnah Sindh Medical University, Karachi, Pakistan; c Ayub Medical College, Abbottabad, Pakistan; d Spinghar University, Kabul, Afghanistan

Global HIV infections have reduced by 35% since 2010; however, new cases have increased among transgender women and cisgender gay and bisexual men, particularly in gender-diverse individuals. Sixty-seven percent of new HIV diagnoses were among cisgender gay men, and above 70% were among Black, Hispanic, or Latino individuals.^[^1^]^ Globally, the use of PrEP remains low, with the achievement of only 16.5% of the UNAIDS goal of 21.2 million users by 2025. Commitment and adoption are especially limited in the most affected groups, emphasizing the need for longer-acting options.

A randomized controlled trial (PURPOSE-1) was carried out in Uganda and South Africa. This study was conducted on cisgender women and young girls between the ages of 16 and 25 years.^[^2^]^ The study population (5,338 women) was previously tested for HIV and determined to be HIV-negative. Gilead Sciences funded and conducted a trial that showed favorable results, achieved positive outcomes, and made lenacapavir accessible to the general population on 25 September 2024.

The participants were divided into three groups in a 2:2:1 ratio for the PURPOSE-1 trial. The first group contained participants who were to receive the lenacapavir injection twice yearly (*n* = 2134); 2136 participants received a daily pill of F/TAF (DESCOVY); and 1068 participants received the F/TDF (TRUVADA) pill daily.

The Purpose 2 trial was a Phase 3, double-masked, randomized controlled trial that compared lenacapavir (every 6 months) to a daily oral F/TDF regimen for HIV prevention. Participants (randomized 2:1) were documented from 92 sites across 7 countries, prioritizing cisgender gay, transgender, and gender non-binary individuals at high risk of HIV. Eligible participants were at least 16 years old, had condomless sex with partners designated male at birth, and had no previous HIV testing or PrEP use.^[^1^]^

For the PURPOSE-1 trial, in the lenacapavir group, no infections were found, with an incidence of 0 per 100 person-years (95% CI: 0.00–0.19). In the F/TAF group, 39 infections were found, with an incidence of 2.02 per 100 person-years (95% CI: 1.44–2.76). In the F/TDF group, 16 infections were found – 1.69 per 100 person-years (95% CI: 0.96–2.74).

Background HIV incidence (population baseline) was 2.41 per 100 person-years (95% CI: 1.82–3.19).^[^2^]^

For Purpose-2, 11 new HIV infections occurred among 3000 participants: 2 in the lenacapavir group (0.10 per 100 person-years) and 9 in the F/TDF group (0.93 per 100 person-years). As compared to the background rate (IRR: 0.04, *P* < 0.001), lenacapavir diminished HIV incidence by 96% and by 86% compared to F/TDF (IRR: 0.11, *P* = 0.002). The two infections in the lenacapavir group had adequate drug levels, and both showed a capsid resistance mutation (N74D). The F/TDF group infections were linked to poor compliance or early discontinuation, with one showing emtricitabine resistance (M184V).^[^3^]^

Similar safety outcomes were noted between groups. Common adverse effects included rectal chlamydia, oropharyngeal gonorrhea, and rectal gonorrhea. Serious adverse effects and discontinuations were rare: 0.2%. Lenacapavir injection-site reactions occurred in 68.8% of cases (vs. 34.9% in placebo injection groups).^[^2^]^ Abnormal laboratory investigations were mostly mild; nevertheless, reduced kidney function was more frequent with F/TDF. Lenacapavir slightly raises the estimated glomerular filtration rate (eGFR). Overall, lenacapavir illustrated high effectiveness and a positive safety profile as a long-term HIV prevention option.

Lenacapavir is a first-in-class, long-acting HIV-1 capsid inhibitor with high efficacy and a long half-life, facilitating twice-yearly subcutaneous dosing.^[^4^]^ It has shown effectiveness in cisgender women and in preclinical models. In the PURPOSE 2 trial, lenacapavir was tested for HIV, with the primary goal being to compare HIV incidence in the lenacapavir group with background population rates; the secondary goal was to compare it to the F/TDF group. The trial followed a design that was actively controlled, avoiding placebo use. In total, 10 094 lenacapavir and 5,145 placebo injections were administered.

Injection-site reactions occurred in 83.2% of lenacapavir recipients versus 69.5% in the F/TDF group, mostly mild or moderate.

Subcutaneous nodules were more frequent in the lenacapavir group (63.4% vs. 39.2%), lasting a median of 183 days. Pain levels were similar between groups, and injection-site reactions decreased over time. Discontinuation due to reactions was low (1.2% lenacapavir vs. 0.3% F/TDF), and no cases of keloid formation were reported.

The HIV-negative individuals who met the eligibility criteria were randomized in a 2:1 ratio to receive either lenacapavir or daily oral F/TDF. The lenacapavir group received 927 mg via two 1.5-ml abdominal injections every 26 weeks (±7 days), along with oral placebo tablets matching F/TDF. They also received oral loading doses of lenacapavir – two 300-mg tablets on days 1 and 2. The F/TDF group received 200 mg of emtricitabine and 300 mg of F/TDF daily.

Twice-yearly subcutaneous lenacapavir has proven its high efficacy in preventing HIV infection among cisgender gay, bisexual, transgender, and gender non-binary individuals, with favorable safety outcomes. Over 99% of lenacapavir recipients remained HIV-free, even with high-risk behaviors such as sexually transmitted infections. These long-term HIV prevention outcomes may improve PrEP uptake.

One of the most important clinical implications is the potential improvement in long-term survival outcomes. By demonstrating significant benefits, this injection offers patients higher effectiveness and safety against the disease.^[^4^]^ The trial also illustrates the importance of multiple and extensive participant enrollment to ensure results apply significantly and promote unbiased access to new HIV prevention methods.

Lenacapavir holds great significance for the future of HIV prevention and treatment due to its unique characteristic as a long-term capsid inhibitor.^[^5^]^ Lenacapavir is already approved for therapy-experienced individuals with multidrug-resistant HIV, and ongoing research is analyzing its incorporation into first-line regimens and as part of injectable therapies. To sum up, lenacapavir represents a major breakthrough in the fight against HIV infection, with the potential to transform both prevention and treatment methodologies globally.

Lenacapavir was generally safe and manageable, with mostly mild-to-moderate injection-site reactions and only 1.2% of discontinuations due to these reactions. Notably, it showed superior potency to F/TDF, even among participants with comparably high responsiveness to daily oral PrEP. The trial highlighted the significance of extensive clinical research concentrating on populations disproportionately affected by HIV. Overall, twice-yearly injectable lenacapavir offers a promising, sustainable PrEP option that could improve access, adherence, and equity in HIV prevention.

## Data Availability

Not applicable.
